# Female Adolescents with Severe Substance and Conduct Problems Have Substantially Less Brain Gray Matter Volume

**DOI:** 10.1371/journal.pone.0126368

**Published:** 2015-05-22

**Authors:** Manish S. Dalwani, Mary Agnes McMahon, Susan K. Mikulich-Gilbertson, Susan E. Young, Michael F. Regner, Kristen M. Raymond, Shannon K. McWilliams, Marie T. Banich, Jody L. Tanabe, Thomas J Crowley, Joseph T. Sakai

**Affiliations:** 1 Departments of Psychiatry, University of Colorado School of Medicine, Denver, CO, United States of America; 2 Departments of Radiology, University of Colorado School of Medicine, Denver, CO, United States of America; 3 Institute of Cognitive Science and Department of Psychology & Neuroscience, University of Colorado, Boulder, CO, United States of America; University of Tokyo, JAPAN

## Abstract

**Objective:**

Structural neuroimaging studies have demonstrated lower regional gray matter volume in adolescents with severe substance and conduct problems. These research studies, including ours, have generally focused on male-only or mixed-sex samples of adolescents with conduct and/or substance problems. Here we compare gray matter volume between female adolescents with severe substance and conduct problems and female healthy controls of similar ages. *Hypotheses*: Female adolescents with severe substance and conduct problems will show significantly less gray matter volume in frontal regions critical to inhibition (i.e. dorsolateral prefrontal cortex and ventrolateral prefrontal cortex), conflict processing (i.e., anterior cingulate), valuation of expected outcomes (i.e., medial orbitofrontal cortex) and the dopamine reward system (i.e. striatum).

**Methods:**

We conducted whole-brain voxel-based morphometric comparison of structural MR images of 22 patients (14-18 years) with severe substance and conduct problems and 21 controls of similar age using statistical parametric mapping (SPM) and voxel-based morphometric (VBM8) toolbox. We tested group differences in regional gray matter volume with analyses of covariance, adjusting for age and IQ at *p*<0.05, corrected for multiple comparisons at whole-brain cluster-level threshold.

**Results:**

Female adolescents with severe substance and conduct problems compared to controls showed significantly less gray matter volume in right dorsolateral prefrontal cortex, left ventrolateral prefrontal cortex, medial orbitofrontal cortex, anterior cingulate, bilateral somatosensory cortex, left supramarginal gyrus, and bilateral angular gyrus. Considering the entire brain, patients had 9.5% less overall gray matter volume compared to controls.

**Conclusions:**

Female adolescents with severe substance and conduct problems in comparison to similarly aged female healthy controls showed substantially lower gray matter volume in brain regions involved in inhibition, conflict processing, valuation of outcomes, decision-making, reward, risk-taking, and rule-breaking antisocial behavior.

## Introduction

Substance use disorders (DSM-IV [1]) and conduct disorder (DSM-IV [1]), strongly comorbid in adolescents [2], are characterized, in part, by the presence of very risky behaviors [3–5] and are associated with severe health [6,7] and economic problems [8,9]. Structural abnormalities in the brain have been reported in adolescents with substance use disorders and conduct disorder [10–13] but most of these studies (including our previous work [11]) have utilized male-only or mixed-sex samples. The goal of this study was to test for structural differences in gray matter (GM) volume in female adolescents with severe substance and conduct problems (SCP) compared to similar aged female healthy controls.

Conduct disorder, while less prevalent in girls than boys, is the second most common psychiatric diagnosis in adolescent girls, with a US adolescent prevalence of about 10% [14]. Conduct disorder is a psychiatric disorder of children and adolescents who show a persistent pattern of behavior in which the basic rights of others, or societal norms or rules, are violated. Conduct disorder in girls is associated with later antisocial personality disorder, early pregnancy, and increased overall mortality [15]. Substance use disorders are also highly prevalent in adolescence and are strongly associated with conduct problems [16–23], and this is true in both genders [24] and in Asian, as well as Western, countries [25]. For example, Crowley et al. (2001) [26] demonstrated that in adolescent patients (n = 87, both genders) the severity of substance problems and the severity of conduct problems strongly correlate (r = .43). The presence of conduct problems in middle childhood is predictive of developing later substance problems [27], suggesting that conduct and antisocial behavior problems are not simply the result of substance-influenced decision making. Such youths with conduct problems are likely to have early onset of substance use [28], multiple substance use disorders diagnoses [2], and persistent courses [29]. Some such youths fail to meet the technical criteria for conduct disorder, because, for example, strictly supervised probation controlled their behavior throughout the last year; therefore, we refer to such youths as having “serious substance and conduct problems” (SCP).

Such diagnostic co-morbidity presents a challenge to research design. Researchers may select a “narrow” or “broad” approach. Researchers selecting the “narrow” approach recruit subjects with only a single diagnosis, excluding all others (e.g., conduct disorder and no other diagnoses). This approach has certain advantages, allowing easy interpretability of findings (e.g., these findings are associated with this disorder). However, there are also some important disadvantages. As stated by Krueger (1999) [30]: “…pure cases of mental disorder (persons meeting the criteria for only one disorder) are not only atypical, they are also less severely impaired. Thus, restricting a study to pure cases limits not only the generalizability of the study, but also the ability to detect the correlates of more severe mental disorders”. A “broad” approach, which we employ here (i.e. selecting cases with both conduct and substance problems), also has advantages and disadvantages. It reduces the ease of interpretation of results, but it does allow recruitment of subjects who are more representative of the source population and are more severely affected.

A strong literature on behavioral disinhibition (BD) further supports that such a “broad” approach to recruitment may have certain advantages. In this literature this conduct disorder and substance use disorder co-morbidity is not treated as a nuisance that must be cleaned. But instead researchers have asked the question: Why do these disorders co-occur within individuals so much more frequently than would be expected by chance? The answer has been because there is a common risk, for both disorders, that has a strong genetic influence. Hicks et al. (2004) [31] in a twin-family study that considered together conduct disorder, alcohol dependence, drug dependence, and antisocial personality disorder (an extension into adulthood of conduct disorder’s antisocial behaviors) concluded that “what parents pass on to the next generation is a general vulnerability to a spectrum of [these] disorders, with each disorder representing a different expression of this general vulnerability” (see also Tully & Iacono, 2014 [23] for a recent review). This common liability sometimes termed behavioral disinhibition (BD) [32–35], goes by many names in the research literature (e.g., neurobehavioral disinhibition [4], externalizing problems [33], and poor self-control [36]). While conduct disorder and individual substance use disorders are moderately heritable [37–42], BD is highly heritable [32–35] and youths may show BD problems early in life, being impulsive, exploratory, excitable, curious, distractible, with less cautiousness, fearfulness, shyness, and inhibition, have difficulty delaying gratification and exhibiting lower levels of self-control [36,43,44]. Youths who show BD problems early in life are more likely to develop substance problems [31,36] and exhibit adult antisocial behaviors [36]. This mounting literature suggests that to understand this common risk for such externalizing disorders we must study their comorbid forms. Adolescents who have antisocial behaviors but no other externalizing behavior problems have only moderate BD; high BD is expressed through the co-occurrence of multiple externalizing behavior problems in a single individual. Therefore, here we recruit adolescents with serious SCP, who are usually individuals with high BD.

Studying structural brain morphometry in adolescent *females* with SCP is important. First, adolescence represents a time of dynamic brain development, especially in regions important to self-control and decision-making [45,46]. Adolescent males and females undergo changes in GM volume across adolescence but at different rates [47], exhibiting sexually dimorphic brain development [48]. Therefore, studying mixed-sex samples may obscure important case-control differences and sex-specific brain differences. Second, although adolescent females try substances of abuse at rates similar to boys, sex differences begin to emerge with greater male rates of substance use disorder prevalence in late adolescence and early adulthood [49]. Similarly, adolescent females compared to males have lower rates of conduct disorder prevalence [14], problems of self-control [50], and risk taking [51]. While these prevalence differences make recruitment of females with SCP more burdensome, some researchers suggest that these phenotypic sex differences may be driven by separate biological or genetic risks in males and females [52,53], encouraging the study of males and females separately.

Although externalizing behavior problems in adolescent females are associated with negative outcomes [15], we find only three studies examining brain morphometry of adolescent female-only samples with SCP or related phenotypes. Fairchild et al. (2013) [54], using region of interest method as their primary analyses, reported lower GM in bilateral insula and right striatum in female adolescents with conduct disorder compared to control females. Fein et al. (2013) [55] showed greater thalamus and putamen volumes in female adolescents with alcohol use disorder versus controls; however, another study on female adolescents with alcohol use disorder versus controls reported smaller prefrontal cortex [13], a brain region critical in inhibition, decision-making, outcome monitoring, and self-evaluation [56]. While the finding of less GM with SCP has been relatively consistent in males [10–13], the relative lack of studies leaves this question unresolved in females.

We previously demonstrated functional and structural deficits using whole-brain analyses in male adolescents with SCP [11,57]. Here we follow that study by comparing a female sample of youths with SCP and controls. The morphometric differences in female adolescents with serious SCP is not known; we therefore constructed our hypotheses based on the broader knowledge gained from the few structural MRI studies focusing on females with conduct disorder and alcohol use disorder [13, 54, 55], the broader literature on the functional neural correlates of inhibition and sensation seeking [23, 57], and on the available literature on males adolescents with SCP [10–13]. However considering there is limited prior work to guide our hypotheses we conducted whole-brain analyses. Hypotheses: Female adolescents with SCP will have less GM compared to controls in frontal lobe regions involved in inhibition (i.e. dorsolateral prefrontal cortex and ventrolateral prefrontal cortex), conflict processing (i.e. anterior cingulate cortex), valuation of expected outcomes (i.e. orbitofrontal cortex) and the dopamine reward system (i.e. striatum).

## Methods

### Ethics Statement

The Colorado Multiple Institutional Review Board approved all procedures. Subjects below 18 provided written informed assent and their parents provided written informed consent. Subjects who were 18 provided written informed consent.

### Inclusion Criteria

Subjects (22 patients and 21 controls) were right-handed females age 14–18 years with estimated Intelligent Quotient (IQ)≥80. Patients were recruited from our university based treatment program for severe SCP as per DSM-IV. Patients had at least one non-nicotine substance use disorder diagnosis.

### Exclusion Criteria

Individuals were excluded if they or their parents lacked sufficient English skills for assenting/consenting, had substances present in urine or saliva about 7 days before and immediately before scanning (urine AccuTest tested for marijuana, cocaine, methamphetamine, amphetamine, barbiturates, benzodiazepines, MDMA, methadone, other opioids, PCP; saliva AlcoScreen for alcohol), or if a urine test for pregnancy was positive. Additional MRI exclusion criteria included obvious psychosis, reported or evidence of marked claustrophobia, orthodontic braces, color blindness, contraindications to MR scanning (e.g., non-MR-compatible devices or implanted foreign bodies), history of head injury with loss of consciousness more than 15 minutes, prior significant neurological illness, or prior neurosurgery. Subjects were not excluded for prescribed medication. Controls had no conduct disorder diagnosis, non-nicotine substance use disorder, court convictions, or substance-related arrests, treatments, or school-expulsions.

### Assessments

Parents completed the Child Behavior Checklist [58] and a socioeconomic status survey [59]. Adolescent subjects completed a 2–3 hour session which included the Diagnostic Interview Schedule for Children (for DSM-IV attention deficit hyperactivity disorder (ADHD) and conduct disorder diagnoses) [60], Composite International Diagnostic Interview Substance Abuse Module (for substance use disorder diagnoses) [61,62], a substance use recency questionnaire, Peak Aggression Score [26], Eysenck Junior Impulsiveness Scale [63], Youth Self Report [26], Carroll Self Rating Scale (for depression) [64], and two subtests (vocabulary and matrix reasoning) from the Wechsler Abbreviated Scale of Intelligence [65].

We operationalized BD as the composite of four measures of disinhibited behavior: lifetime conduct disorder symptom counts, lifetime substance use disorder symptom counts (i.e., lifetime substance abuse and dependence symptom counts), a subset of Child Behavior Checklist items counts (or Youth Self Report for 3 subjects with missing Child Behavior Checklist) for inattention and hyperactivity/impulsivity. Subjects' scores were scaled against a sample of 414 similarly aged, similarly-assessed, community female adolescents and those scores were weighted and summed yielding a Z-score of BD relative to that community sample (for details see http://ibgwww.colorado.edu/cadd/bd.html).

### Image Acquisition

We acquired high-resolution 3D SPGR-IR T1–weighted coronal image of all the participants from a 3T GE MR scanner using the following parameters: TR/TE/T1/Flip = 9ms/1.9ms/500ms/10°, FOV = 22^2^ in plane, slice thickness = 1.7mm, 256^2^ matrix, number of slices = 124. Total scan time was 9 minutes, 12 seconds.

### Data Analyses

#### Patient-Control Differences: demographic, diagnostic, self-report, and behavioral data

We used statistical tests as appropriate (including t-test, chi-square, Mann-Whitney, and Fisher-Exact) to compare demographic and clinical measures including: age, race, and socio-economic status, cognitive measured IQ, number of substance dependence symptoms, substance use disorder diagnoses, and lifetime conduct disorder diagnosis. We also compared groups’ scores for ADHD, depression, aggressiveness, impulsivity, and BD.

#### Patient-Control Differences—GM Volume

We used an in-house Java (www.java.com) program to convert Dicom images into 3D analyze format and statistical parametric mapping (SPM8; The Wellcome Trust Centre for Neuroimaging at University College London) and Matlab (R2012a) (Mathworks Inc, Natick, MA, USA) software for data analyses. Structural images were excluded if motion artifacts were seen.

Within SPM8, we used the Template-o-matic (TOM8; http://dbm.neuro.uni-jena.de/software/tom/) toolbox to produce tissue probability maps (TPM) specific to our adolescent female sample. In combination with age and sex data from our sample, the TOM8 toolbox draws from the Pediatric MRI Data Repository created by the NIH MRI Study of Normal Brain Development (http://pediatricmri.nih.gov/nihpd/info/index.html) to create study-specific TPM [66]. We then utilized these TPM files for brain segmentation into GM, white matter (WM), and cerebral-spinal fluid (CSF) through the voxel-based morphology (VBM8) toolbox. VBM8 implements these segmented images in the Diffeomorphic Anatomical Registration Through Exponentiated Lie Algebra (DARTEL) algorithm. DARTEL is a high-dimensional spatial normalization technique that utilizes the segmented images to produce six templates (GM, WM, CSF, bone, air and skull). Finally, the images were registered using the final DARTEL templates to provide modulated GM and WM.

The term modulation means multiplying each voxel intensity by the Jacobian determinant, which characterizes how much a voxel was stretched or contracted during normalization. Images were modulated to preserve accurate representation of volume. The modulated GM images were smoothed with a 8mm full-width half maximum Gaussian filter.

We compared volume estimates between groups using whole-brain voxel-wise analyses of covariance (ANCOVAs), adjusting for age and IQ at *p*<0.05, corrected for multiple comparisons at whole-brain cluster-level threshold and voxel-wise *p*<0.005. Cluster threshold corresponded to 395 voxels or 1333.1 mm^3^ (since each voxel is 1.5 mm^3^). Unlike the method used in the male adolescent sample for our previous publication [11], we did not include total gray volume as a covariate as the new segmentation methods and modulation intrinsically account for global brain size differences.

Using the region of interest analyses toolbox called “MARSBAR” [67], we extracted mean GM volume for each subject for any region showing a patient-control difference and illustrated histograms for each group. MARSBAR extracts simple mean GM volumes, i.e. averages were unadjusted for any variables in the SPM model.

#### Secondary Analyses

Using these average GM volumes for the 9 clusters (or regions of interest (ROIs)) estimated from MARSBAR, a ROI approach was implemented for all the secondary analyses. Each ROI and the total GM volume (for total 10 measures) served as separate dependent variables in ANCOVAs fit in SAS v9.4 (http://support.sas.com/documentation/cdl/en/statug/63033/HTML/default/viewer.htm#statug_glm_sect049.htm) as follows:

1. Because ADHD and depression are often comorbid with conduct disorder and substance use disorders [2], we evaluated group effects in ANCOVAs adjusting for age, IQ, ADHD and depression scores.
2. To insure that our group differences were not superseded by interactions with age or IQ (i.e. lack of parallelism), we evaluated group effects in ANCOVAs adjusting for age, IQ, and the group by age and group by IQ interactions.
3. Finally to insure that our group differences were not driven by any medication use (see S1 Table in the supporting information section), we evaluated group effects in ANCOVAs adjusting for age and IQ after removing the 5 controls and 9 patients who reported being on medication (at the initial off-site interview as shown in S1 Table).

#### Exploratory analyses within patients

After examining and testing the primary and secondary aims, we evaluated whether GM volume was related to certain behavioral constructs (or symptoms) in patients only, using exploratory analyses. Specifically, using whole-brain voxel-wise regression analyses adjusting for age and IQ, we tested associations between (1) GM volume and recency of non-nicotine substance use, (2) GM volume and conduct disorder symptom count (z scores), and (3) GM volume and a composite construct for BD. The basic framework in regression analyses within patients consisted of GM volume as the dependent variable and, age, IQ, and the covariate of interest (*e*.*g*., BD, conduct disorder, etc.) as independent variables. We then tested the voxels that showed significant positive or negative associations with covariate of interest after adjusting for nuisance covariates age and IQ. Finally the t-map was transformed to correlation coefficient (r) (http://dbm.neuro.uni-jena.de/vbm/threshold-and-transform-spmt-maps/).

We used the same whole-brain cluster-level and voxel-level statistical thresholds throughout all analyses to maintain consistency.

## Results

Table 1 presents patient-control comparisons. As expected, patients had significantly more conduct disorder and substance use disorder symptoms. In addition, patients reported significantly greater impulsivity, aggression, depression, and BD. With regards to demographic variables, controls had obtained a significantly higher level of education and had significantly higher IQ

**Table 1:** Patient-Control Comparisons on Demographics, Diagnostic and Behavioral Measures.

		Controls (n = 21)	Patients (n = 22)	p-value
**Demographics:**				
Age^1^ (mean years (sem))		16.67 (0.25)	16.09 (0.20)	0.08
IQ^1^ (mean score (sem))		103.95 (2.26)	94.26 (2.23)	**0.0044**
Race	Caucasian (n)	13	12	
	African American (n)	1	1	
	Hispanic (n)	1	7	
	Other (n)	6	2	
	Caucasian vs. Non-Caucasian^2^			0.62
Education	Highest grade completed^3^	10.00 (0.30)	8.77 (0.17)	**0.002**
Socioeconomic Status^1^ ^a^		36.14 (3.57)	45.19 (3.34)	0.08
**Diagnostic and other measures:**				
Eysenck Impulsivity Scale^1^		5.62 (1.00)	14.68 (1.23)	**<0.0001**
Attention Deficit Hyperactivity Problem score^3^ (CBCL or YSR (n = 6))^b^		1.48 (0.40)	5.68 (0.81)	**<0.0001**
Aggression^2^ (n^c^)		0/21	21/22	**<0.0001**
Carroll rating of depression^1^		4.33 (0.77)	10.95 (1.23)	**<0.0001**
No. of substance dependence symptoms^3^		0.24 (0.24)	13.09 (1.66)	**<0.0001**
Lifetime DSM-IV based conduct disorder diagnosis (n)^2^		0/21	14/22	**<0.0001**
Lifetime DSM-IV based substance use disorder diagnosis (n)				** **
	Alcohol^2^	0/21	19/22	**<0.0001**
	Amphetamine^4^	0/21	4/22	0.11
	Cannabis^2^	0/21	20/22	**<0.0001**
	Club Drugs^4^	0/21	10/22	**<0.0005**
	Cocaine^4^	0/21	4/22	0.11
	Hallucinogens^4^	0/21	1/22	1
	Tobacco^4^	0/21	10/22	**0.0005**
Recency of use (mean days before scan (sem)) ^d^ ^e^ ^f^		NA	73.37 (16.54)	NA
Length of Substance dependence (mean years (sem))		NA	1.53 (0.29)	NA
Behavioral Disinhibition (BD Z-score) (sem))^3^		-0.34(0.12)	4.92 (0.56)	**<0.0001**

Significant differences are presented in bold font.

Abbreviations: sem: standard error of mean; NA: Not Applicable; CBCL: Child Behavior Check List

YSR: Youth self report; IQ: Intelligence Quotient

DSM: Diagnostic Statistical Manual

^1^t-test

^2^chi-square

^3^Mann-Whitney

^4^Fisher-Exact

^a^Socioeconomic status was unavailable on 6 patient families

^b^For ADHD score, if parent CBCL not available (n = 6), YSR was used.

^c^For aggression score, controls (means (sem)) = 0(0) and Patients (means (sem)) = 5.73(0.55)

^d^Recency use data is not available on the first three patients due to late addition to the instrument battery

^e^Range of non-nicotine substance use in patients: 8–230 days before scan

^f^ nicotine use in the 18 patients with recency use data (range 5–1440 hours; i.e. <1 day-60 days

note the maximum range is right censored i.e., 1440 hours is the maximum time recorded)

### Whole-Brain Analysis

**Fig 1:**
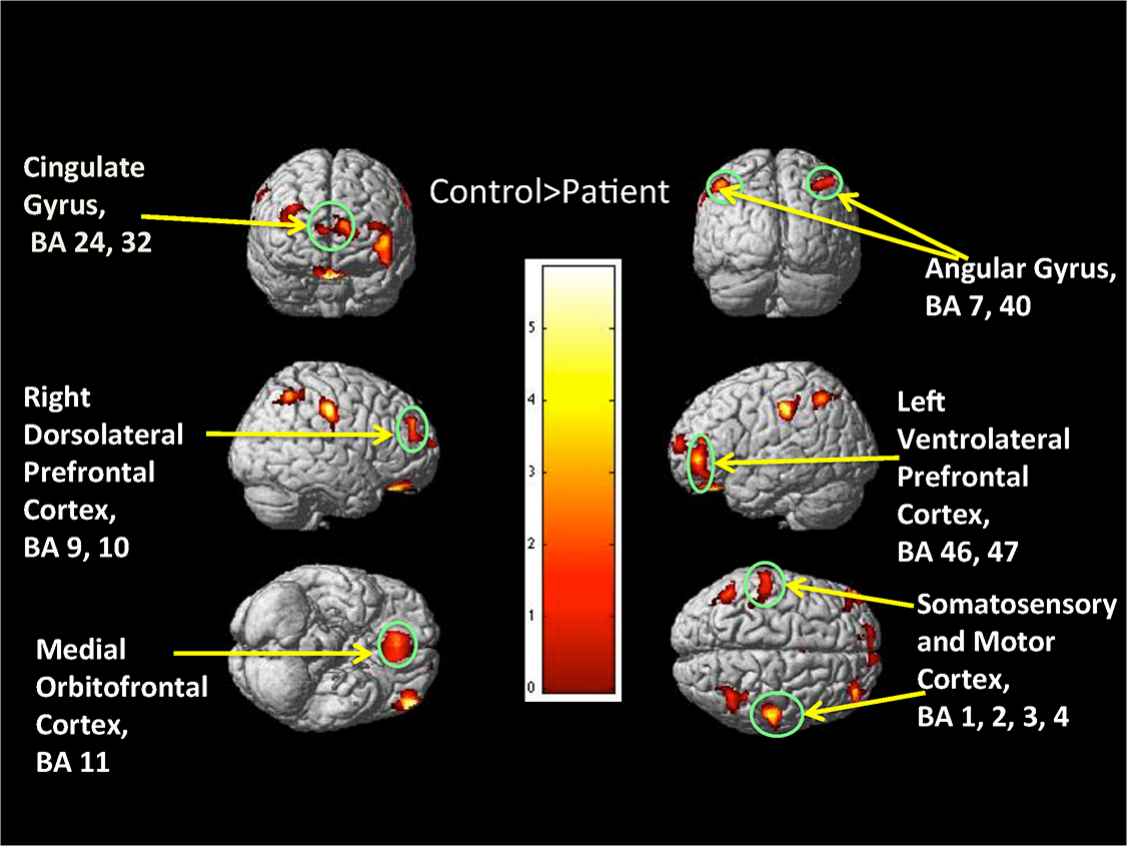
Whole brain VBM analyses: Controls>Patients. The 3D color map showing various frontal and parietal regions where Controls>Patients. Color bar represents t-value.

As shown in Table 2, and Fig 1, VBM analyses demonstrated that patients had less GM volume than controls in several regions: i) medial prefrontal cortex, ii) left ventrolateral prefrontal cortex, iii) right dorsolateral prefrontal cortex, iv) medial orbitofrontal cortex, v) bilateral anterior cingulated cortex, vi) right somatosensory and motor cortex, vii) left somatosensory and motor cortex and supramarginal gyrus, viii) right angular gyrus, and ix) left angular gyrus. Table 2 provides specific details about significant gyri and Brodmann areas (BA). There were no regions where patients had significantly greater GM volume than controls. Whole brain overall GM volume was also significantly lower in patients compared to controls (*p* = 0.035).

**Table 2:** Whole brain analysis results where female controls showed greater GM volumes than SCP female patients.

Region	Laterality/BA	# Voxels	t*	x	y	z
**Controls>Patients**						
**Medial Prefrontal Cortex**		959	3.6	-15	43	12
Sup. Fr. Gy.	R,L 10					
Med. Fr. Gy.	R,L 10					
ACC	L 32					
Mid. Fr. Gy.	L 9					
**Left Ventrolateral Prefrontal Cortex**		1353	4.8	-46	49	3
Inf. Fr. Gy.	L 11, 46, 47					
Mid. Fr. Gy.	L 10, 47					
**Right Dorsolateral Prefrontal Cortex**		560	3.5	33	48	30
Sup. Fr. Gy.	R 9, 10					
Mid. Fr. Gy.	R 9, 10					
**Medial Orbitofrontal Cortex**		1518	3.0	37	-30	4.9
Rectal Gy.	R,L 11, 47					
Orbital Gy.	R,L 11, 47					
**Cingulate Gyrus**	R,L 24, 32	721	4.2	-13	3	43
**Right Somatosensory & Motor Cortex**		995	5.8	52	-19	42
Postcentral Gy.	R 1, 2, 3					
Precentral Gy.	R 4					
**Left Somatosensory & Motor Cortex & Supmarginal Gyrus**		1274	4.0	-43	-28	36
Postcentral Gy.	L 1, 2, 3					
Precentral Gy.	L 4					
Inf. Parietal Lobule	L 40					
**Right Angular Gyrus**		721	4.0	39	-55.0	53
Inf. Parietal Lobule	R 40					
Sup. Parietal Lobule	R 7					
**Left Angular Gyrus**		495	4.4	-48	-63	54
Inf. Parietal Lobule	L 40					
Sup. Parietal Lobule	L 7					
**Total # of Voxels**		8596				

* t-value of the voxel with maximum GM difference in the cluster. The x,y,z co-ordinates represent the Montreal Neurological Institute (MNI) space location of the voxel with maximum GM difference.

**Abbreviations:** ACC: Anterior cingulate cortex; BA: Brodmann area; Fr: Frontal; Gy: Gyrus; Inf: Inferior frontal gyrus; L: Left; Med: Medial; Mid: middle; R: Right; Sup: Superior

### Analysis of clusters showing patient-control differences

**Fig 2:**
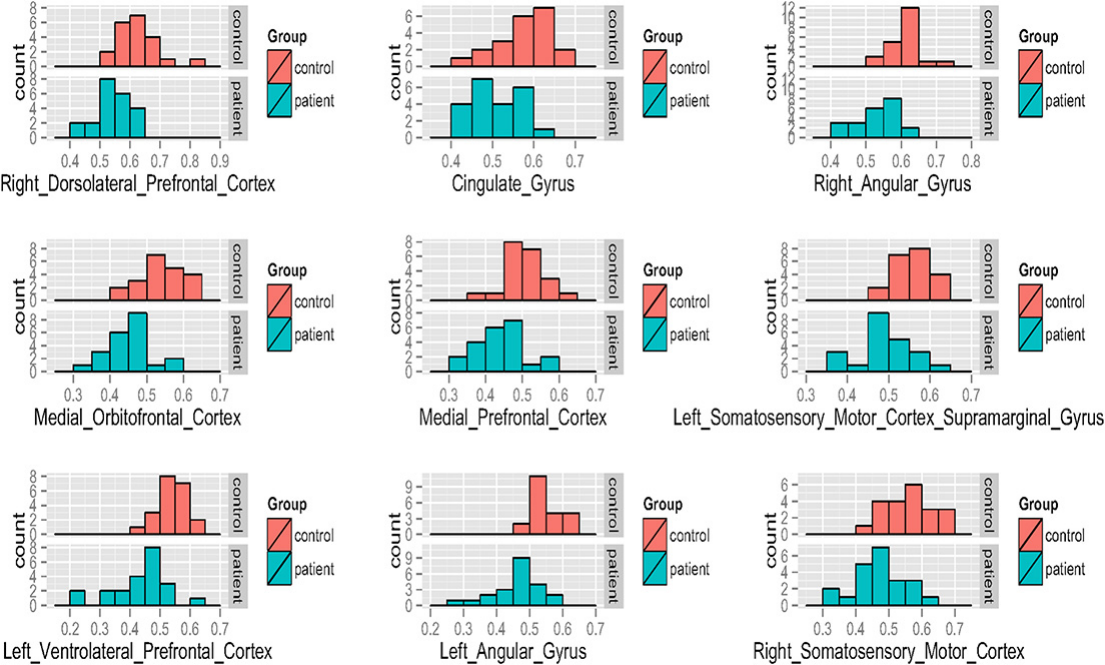
GM volume details of ROI where Controls>Patients. Nine panels which correspond to the clusters of Control>Patient GM volume demonstrated with whole-brain analyses. Each panel displays two histograms, salmon color bars indicating controls and teal color bars indicating patients. For all histograms, the x-axis represents GM volume (intensities mapping GM volume in mL) and the y-axis represents number of subjects.

Fig 2 illustrates histograms for each group of the MARSBAR-extracted-per-subject GM volume from the nine clusters (or regions) of significant group differences at the whole-brain analyses. The distributions of GM volumes for patients and controls show group mean differences but clear overlap for all clusters. Table 3 provides within-group MARSBAR-extracted-per-subject GM volume means, standard deviations, and percent difference in GM volume between groups for each cluster.

**Table 3:** ROIs where GM volumes in female controls exceeded GM volumes in SCP patients.

	Controls (n = 21)	Patients (n = 22)	Reduction (%)		
Significant Cluster (from the whole-brain analyses)	(means±sd)	(means±sd)	Patients	p-value*	p-value**
**Cingulate Gyrus**	0.57±0.06	0.50±0.06	12.28	0.0007	0.0009
**Medial Prefrontal Cortex**	0.51±0.05	0.44±0.07	13.73	0.001	<0.0001
**Left Ventrolateral Prefrontal Cortex**	0.54±0.05	0.43±0.10	20.37	0.0008	0.002
**Right Dorsolateral Prefrontal Cortex**	0.63±0.07	0.55±0.05	12.70	0.0005	0.0002
**Medial Orbitofrontal Cortex**	0.54±0.05	0.46±0.06	14.81	0.0002	0.0001
**Left Somatosensory Motor Cortex**	0.56±0.04	0.49±0.06	12.50	<0.0001	0.0003
**Left Angular Gyrus**	0.54±0.06	0.46±0.07	14.81	0.0005	0.004
**Right Angular Gyrus**	0.61±0.04	0.53±0.06	13.11	<0.0001	0.0001
**Right Somatosensory Motor Cortex**	0.56±0.07	0.48±0.07	14.29	<0.0002	0.004
**Total GM Volume (ml)**	685.14±52.28	620.29±77.84	9.47	0.035	0.01

The table presents GM volume in each group for ROIs where controls>patients. The table also includes total GM volume for each group.

*Analyses of covariance: ROI as dependent variable and group, age and IQ as covariates.

**Analyses of covariance: ROI as dependent variable and group, age, IQ, depression and ADHD as covariates. The units for the 9 ROIs (clusters) are SPM units, which map intensities to gray matter volume (mL).

### Secondary analyses

Testing whether ADHD and Depression influenced our primary results: ANCOVAs adjusting for age, IQ, ADHD, and depression scores showed significant patient-control differences in mean GM volume for all nine ROIs and also for total GM, supporting that the results remain significant (*p*<0.05) between patient and controls even after accounting for ADHD and depression (Table 3).

Testing whether a lack of parallelism influenced our primary results: ANCOVAs adjusting for age, IQ, Group, Group x Age and Group x IQ for each of the nine ROIs (and also total GM volume) showed no significance for the interaction coefficients, suggesting that the group effects were not superseded by any interactions with age and IQ.

Testing whether medication use influenced our primary results: ANCOVAs adjusting for age and IQ continued to show group differences for all nine ROIs and total GM volume after removing the medicated subjects (5 controls and 9 patients) (see S2 Table in the supporting information for details)

### Exploratory regression analyses within patients

#### GM and recency of non-nicotine substance use

Whole-brain regression analysis within patients showed no regions significantly related to recency of non-nicotine substance use.

#### GM and BD

**Fig 3:**
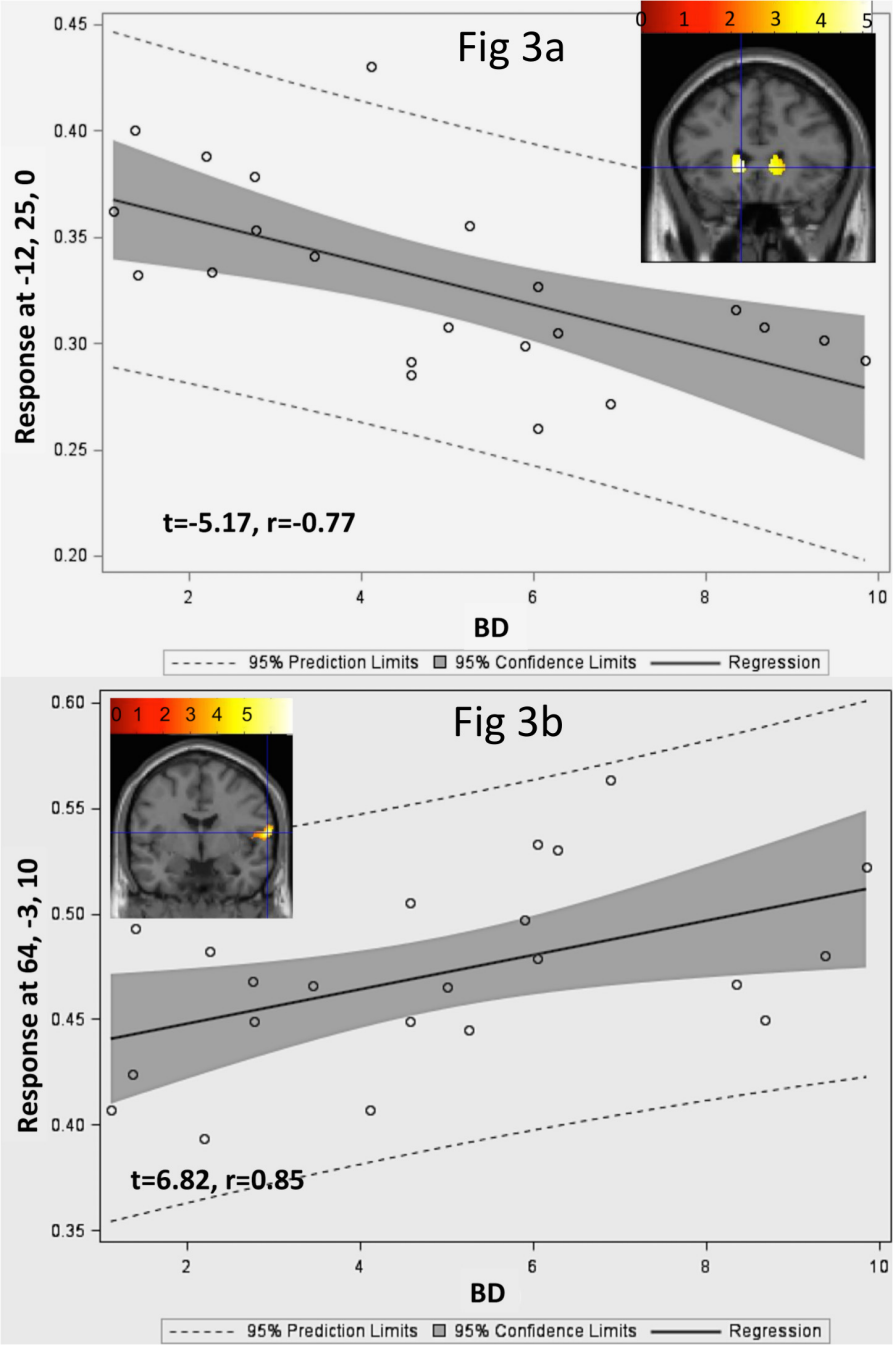
Whole brain regression analyses with BD within patients. Color map (Y coordinate: 25 mm) in Fig 3a showing negative association between the bilateral striatum and BD score in patients after co-varying for age and IQ. Color map (Y coordinate = -3 mm) in Fig 3b showing positive association between the insula and BD score in patients after co-varying for age and IQ. The crosshairs mark the voxel with maximum association and the regression plots below the color maps show the association between the region and BD score for that voxel with maximum association. A 95% confidence interval will contain the true parameter (slope) with probability 0.95. The prediction interval is an estimate of an interval in which future observations will fall, with probability 0.95, given what has already been observed. Rt = Right.

Whole-brain regression analysis within patients showed a significant negative association between BD and bilateral striatal GM volume (including caudate, putamen and nucleus accumbens; left cluster size: 539 voxels and right cluster size: 471 voxels), suggesting that higher BD was associated with smaller striatal GM volume. Fig 3A shows the strong negative association between striatum cluster and BD and the regression line at the voxel with maximum level of association in the left striatal cluster (t = -5.1, r = -0.77). Whole-brain regression analysis within patients showed a significant positive association between BD and GM volume in the insula cluster, (BA 13), that extends to superior temporal gyrus (BA 22), postcentral gyrus (BA 43), and supramarginal gyrus (BA 40) (cluster size: 2088 voxels). Fig 3B shows the strong positive association between the insula cluster and BD and the regression line at the voxel with maximum level of association in the right insula cluster (t = 6.82, r = 0.85).

Note, our whole-brain comparison did not show any group differences in the striatum. But because we utilized a cluster threshold criterion in our analyses, even highly significant differences in small regions would be missed. We therefore extracted GM volume for each subject from the striatum clusters that our exploratory analyses suggested had a negative association with BD in patients. We then tested post hoc if there were any group differences in the two striatum clusters (left and right) after adjusting for age and IQ (see S1 Text for more details) and found no significant group differences in the striatum.

#### GM and Conduct Disorder

**Fig 4:**
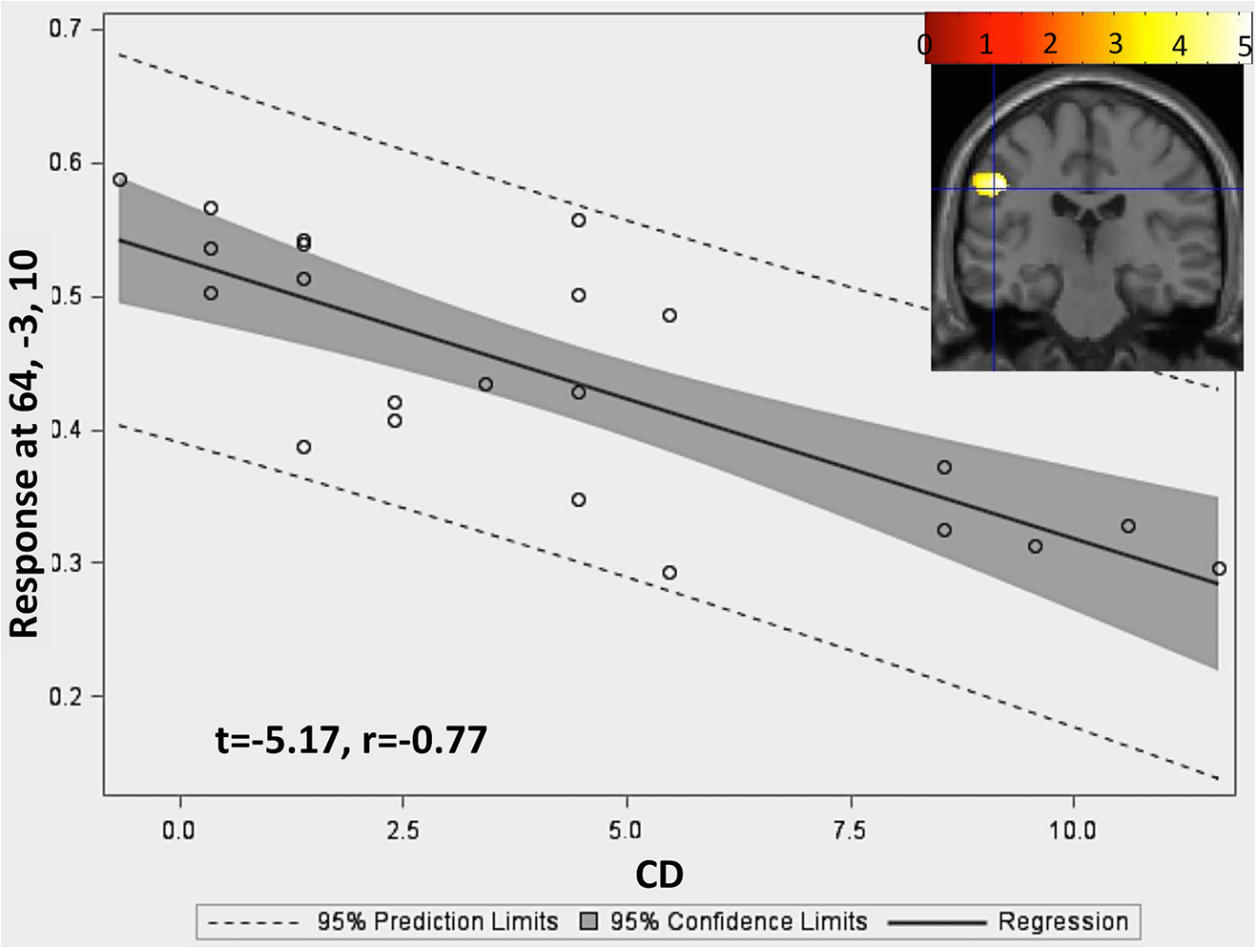
Whole brain regression analyses with conduct disorder symptom count within patients. Color map (Y coordinate: -25 mm) showing negative association between the left somatosensory and motor cortex region (i.e. precentral and postcentral gyrus (BA 1, 2, 3 and 4)) and supramarginal gyrus and inferior parietal lobule (BA 40)) and BD score in patients after co-varying for age and IQ. The crosshair marks the voxel with maximum association and the regression plots below the color map shows the association between the region and conduct disorder symptom count Z-score for that voxel with maximum association. Rt = Right.

Whole-brain regression analysis within patients showed a significant negative association between conduct disorder symptom count and GM volume in one frontoparietal cluster (size: 634 voxels; cluster description: left somatosensory and motor cortex region, supramarginal gyrus, and inferior parietal lobule). Fig 4 shows the strong negative association between this frontoparietal cluster and conduct disorder and the regression line at the voxel with maximum level of association (t = -5.1, r = -0.77).

## Discussion

The goal of this study was to examine structural GM differences between female adolescents with SCP and similar aged female controls; we extend the limited literature available [54] by presenting whole brain structural analysis comparing adolescent females with severe externalizing behavior problems to healthy controls, though prior work has focused on related phenotypes, namely conduct and alcohol use disorders. We demonstrate widespread regions of smaller GM volume in female adolescents with SCP, especially in frontal and parietal regions, and 9.5% smaller overall whole brain GM volume compared to controls.

In frontal lobes, patients showed in all 5 clusters identified in whole-brain analyses more than 10% smaller GM volumes than controls (See Table 3; largest GM difference was observed in ventrolateral prefrontal cortex). The prefrontal cortex covers 29% of the total cortex and plays a central role in executive function and goal-directed behavior [68]. BA11, BA24, BA25 form the ventral prefrontal cortex and are involved with behaviors associated with reward and emotions; BA32, BA9 and BA44 form the dorsal prefrontal cortex and are involved with inhibitory control, working memory, and appraisal of errors; BA11, BA25 form medial prefrontal cortex and play a role in salience attribution [68]. In the past, we have reported functional widespread hypoactivation in prefrontal cortex in males with SCP [57]. Smaller GM volumes observed here in anterior cingulate cortex (BA32) and dorsolateral prefrontal cortex (BA8, BA9, BA10) may reflect dysfunction and result in poor decision-making, impairment in internal awareness, problems with error detection, and disinhibited antisocial and drug-using behavior [36,47]. Similarly, smaller GM in ventromedial prefrontal cortex (BA11, BA25) could reflect disrupted inhibitory control in individuals with substance use disorder leading to impulsivity and poor control over behavior [68]. Our results are consistent with the growing literature on functional and structural brain differences in adults with antisocial and substance use disorders implicating deficits in frontal regions [69].

As for parietal regions, females with adolescent SCP showed in all clusters more than 10% reductions in GM volumes, compared with controls (see Table 3). Frontoparietal networks likely play important roles in executive function and decision-making [70]. For example, the angular gyrus activates in moral judgment tasks, is a key region in individuals with antisocial problems, and has been implicated in rule-breaking behavior [71]. Smaller angular gyri observed here in patients may reflect a neuroanatomical locus for this component of the behavioral pathology.

Although our patients had significantly higher ADHD and depression scores, the patient-control differences were largely unaffected by controlling for ADHD and depression (along with age and IQ) in our secondary analyses. Thus, our secondary analyses support that our clusters exhibiting patient-control differences were not driven by differences in these comorbid disorders. Our secondary analyses also provide evidence that our primary results showing group differences in GM volumes were not driven by medication effects nor invalidated by interactions of group with age or IQ.

Our design is cross sectional and our “broad” approach to subject recruitment regarding co-morbidity limits the ability to absolutely link findings with one disorder or another. Our study was mainly designed to evaluate group differences and thus, brain morphometry associated with SCP in adolescent females. However, we considered it important to investigate (through regression analyses) whether particular diagnoses or traits were associated with one finding vs. another. These exploratory analyses should of course be interpreted with caution but can provide useful information for hypothesis generation in future studies. One limitation of our cross-sectional design is that it cannot determine if these clusters of smaller GM volumes represent lower GM as an effect (i.e., atrophy from repeated drug exposure), pre-existing GM deficits as a cause (i.e., differences that pre-date drug exposure), or some combination thereof. Thus, we conducted exploratory analyses to further characterize the relationship between structure and behavior. First, we examined within-patients whether GM volume was associated with recency of drug use. If drug exposure leads to the GM volume loss and such loss improves with abstinence as has been suggested by some adult studies [72], we would expect GM volumes to increase with length of abstinence from drugs of abuse. In contrast to this expectation, these regression analyses failed to produce any positive or negative associations between GM volume and recency of drug use.

Second, we sought to test whether GM volume was associated with BD. Given that BD has been conceptualized as a pervasive and highly heritable trait [35] that predicts the development of substance use disorder [4], strong associations of GM volume with BD might suggest a predisposing neural substrate for substance use disorder. Interestingly, those within-patient analyses demonstrated that higher BD scores are associated with smaller GM volume in striatum. The striatum and nucleus accumbens play a critical role in brain reward circuitry implicated in the craving properties of drugs of abuse [73]. One study of a large community sample of healthy adolescents (n = 226) found that smaller striatal volumes are associated with greater risk taking [74], a major feature of BD. Another group reported that problems of striatal sensitivity are related to problems of behavioral controls and predicts substance use problems later in life [75]; the higher BD association with smaller striatum observed in our study expands our understanding of this functional relationship, demonstrating this structure-behavior relationship in a clinical population, although it remains to be seen if this association predates pathology. The insula has been implicated in empathic processing, and increased insula GM volume has been reported in boys with conduct problems and callous-unemotional traits compared to boys without these problems [76]. Our study extends this finding, reporting a positive correlation between BD and insular GM volume in female SCP. Finally, despite our reasons for recruiting a sample of female adolescents with high BD, we recognize that some brain findings may be specific to one disorder or another. Therefore, to test whether some of our findings were specific to conduct disorder, we conducted regression analyses within patients exploring the relationship between GM and lifetime conduct disorder symptom count. These analyses demonstrated one cluster in supramarginal gyrus where GM volume negatively correlated with conduct disorder symptoms; this cluster approximately overlapped with one of the clusters in the patients-versus-controls whole-brain comparison. Thus smaller GM volume in this supramarginal gyrus (BA 40) cluster may be more specific to conduct disorder and not BD or substance use disorder.

Although, we see substantial lower GM in the inhibition system of female adolescents, we did not see any group difference in the striatum (also see S1 Text). Our exploratory analyses demonstrated a negative association between patient’s striatum GM volume and BD. Tully and Iacono (2014) [23] suggested that individuals with comorbid externalizing disorders suffer both from a hyperactive (increased activation) dopaminergic reward system and a dysfunctional hypoactive (diminished activation) inhibition system. They further attribute individuals with substance use disorders to have more problems in the reward circuitry and individuals with antisocial problems to have more problems in the inhibition system. In contrast Fairchild et al. (2011) [54] showed GM difference in the striatum in female adolescents with conduct disorder. A longitudinal prospective design is needed to examine the developmental brain morphometry associated with antisocial and drug problems.

Our results must be viewed within the context of the following study limitations: (1) Our design cannot determine if smaller GM volumes in female adolescents with SCP antedate drug use. However, our analyses do provide support that adolescent females, with an average of only a few years (1.53 ± 0.29) of heavy drug involvement have widespread regions of smaller GM volume in several frontoparietal clusters. (2) Our study focuses on adolescent females, and the results cannot be extrapolated to adolescent males though we can compare them to our previous study. Relative to our results in adolescent males [11], females with SCP appear to show strikingly more widespread GM changes relative to controls. (3) Several of our subjects were on medications, and medication effects cannot be absolutely ruled out. However, our analyses after removing subjects on medication (S2 Table) were consistent with our primary findings.

## Conclusions

Comorbid substance use disorder and conduct disorder are major economic and societal concerns, often causing great harm to those individuals suffering with these disorders. We demonstrate that adolescent females with SCP, after only a few years of heavy substance use, show smaller GM volumes than controls across broad cortical regions, including some that are associated with decision-making, conflict processing, evaluation of outcomes, and inhibition. These results are a critical first step but future longitudinal work to both monitor youths from a young age and follow adolescent females with SCP during prolonged abstinence could help in understanding the pre-existing neuronal structural vulnerability to SCP, the adverse effects of chronic drug use, and whether substance-induced GM volume changes remit with abstinence. If a causal link between substance use and GM volume loss can be established, that information could be incorporated into prevention efforts with younger children. If substance-induced GM volume changes remit with abstinence, such information might be utilized to enhance motivation of SCP adolescent females in treatment.

## Supporting Information

S1 Text Examination of striatum GM volume difference between controls and patients. (PDF)

S1 Table List of subjects who were prescribed some form of medication at the initial interview and at the time of the MRI. (PDF)

S2 Table Region of interest analyses without the medicated subjects. The region of interest (ROIs) where GM volumes in female controls exceeded volumes in SCP patients after excluding subjects on medication.
(PDF)
