# Supplementary material for: Female Adolescents with Severe Substance and Conduct Problems Have Substantially Less Brain Gray Matter Volume
Source: PLoS One. 2015 May 22;10(5):e0126368. doi: 10.1371/journal.pone.0126368 (PMC4441424; doi:10.1371/journal.pone.0126368)
Supplement: S1 Text — (PDF) [file pone.0126368.s001.pdf]

Supporting information S1 Text: Striatum difference between groups.

Exploratory analyses within patients showed a significant negative association between striatum and BD. However, the whole brain analyses conducted with the family wise error corrected threshold did not show any striatal GM volume difference between groups even though patients had significantly higher BD than controls. To examine post-hoc if there were any striatum differences by group that were missed due to the conservative threshold, we extracted for each subject using MARSBAR ROI toolbox the GM volume for the striatum clusters (left and right separately) that were observed in the regression analyses within patients. These GM volumes were unadjusted for the variables in the SPM model. We then compared groups on the extracted striatum GM volumes after adjusting for age and IQ and found no significant differences. We then correlated the BD scores with GM estimates for right and left striatum for both controls and patients and plotted the correlations as shown below in S1a and S1b Figs:

S1a Fig: Behavioral Disinhibition score vs. Left Striatum GM volume.

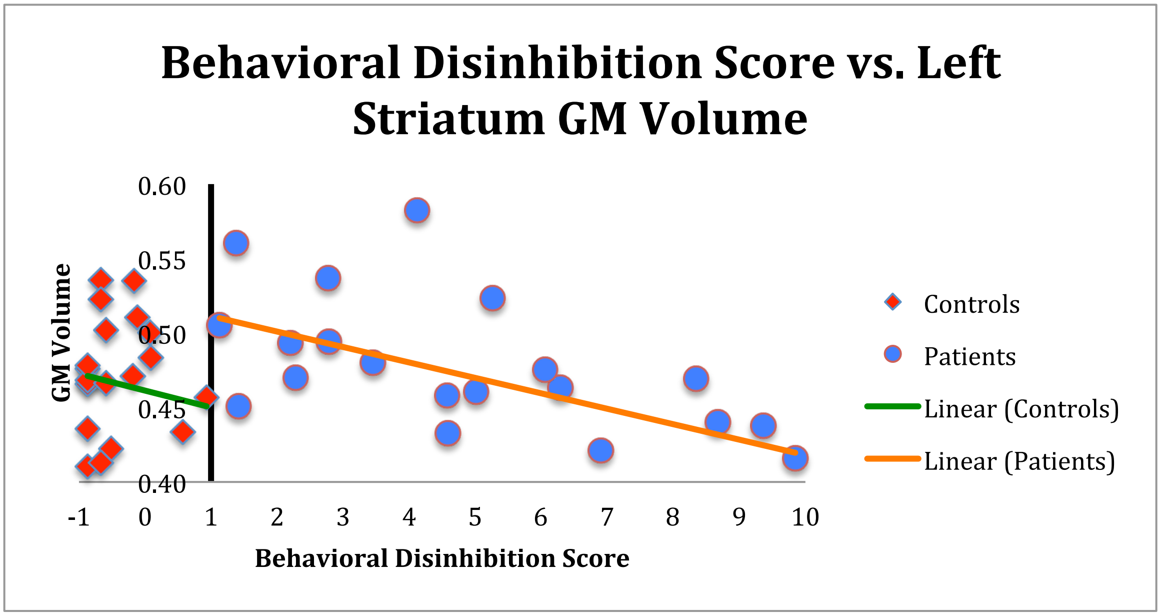

S1b Fig: Behavioral Disinhibition score vs. Right Striatum GM volume.

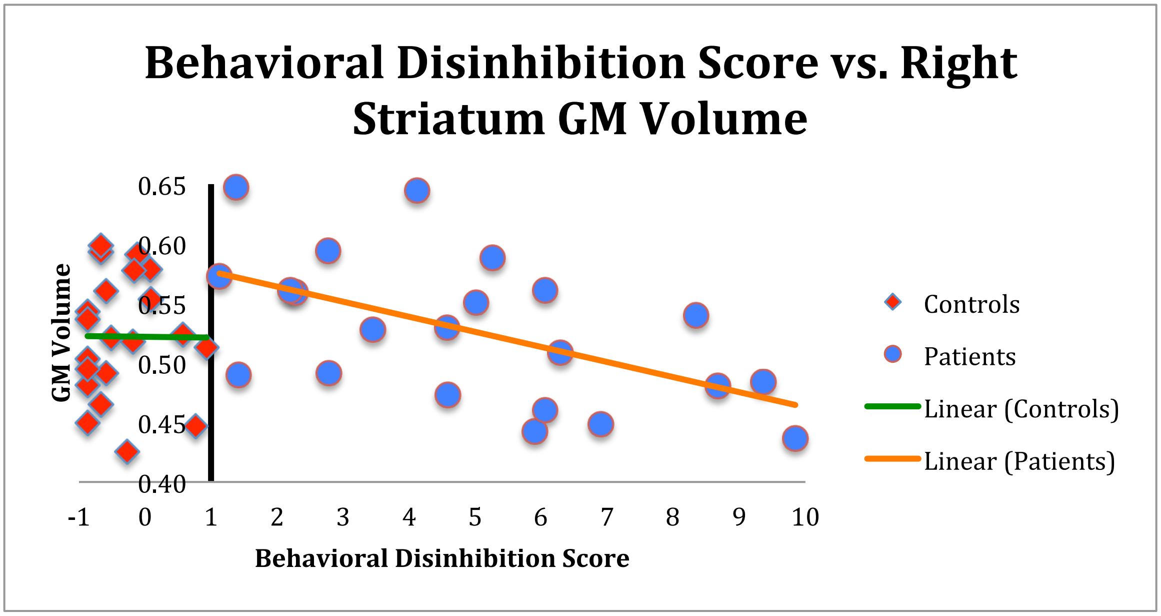

The patients as expected from the whole-brain analyses showed a significant negative correlation (r=-0.55) for average left striatum GM vs. BD Z-score and a significant negative correlation (r=-0.54) for average right striatum GM vs. BD Z-score. The correlations although negative were very weak and non-significant in controls. The main reason for this was the lack of variability in BD scores in controls as shown in the correlation figures above.
