# Supplementary material for: Female Adolescents with Severe Substance and Conduct Problems Have Substantially Less Brain Gray Matter Volume
Source: PLoS One. 2015 May 22;10(5):e0126368. doi: 10.1371/journal.pone.0126368 (PMC4441424; doi:10.1371/journal.pone.0126368)
Supplement: S1 Table — (PDF) [file pone.0126368.s002.pdf]

Supporting Information S1 Table: List of subjects who were prescribed some form of medication at the initial interview and at the time of the MRI.				
**Subjects**	**On Medication at Initial Interview (0=No, 1=Yes)**	**If Yes, Name of medication**	**On Medication within 24 hrs of MR scan (0=No, 1=Yes)**	**If Yes, Name of medication**
control 1	0	N/A	Not asked*	N/A
control 2	0	N/A	Not asked*	N/A
control 3	1	Fluoxetine	Not asked*	N/A
control 4	0	N/A	Not asked*	N/A
control 5	1	Lansoprazole, Spironolactone	1	Lansoprazole, Spironolactone
control 6	0	N/A	0	N/A
control 7	0	N/A	0	N/A
control 8	0	N/A	0	N/A
control 9	1	Ibuprofen	0	N/A
control 10	0	N/A	0	N/A
control 11	0	N/A	0	N/A
control 12	0	N/A	0	N/A
control 13	0	N/A	0	N/A
control 14	0	N/A	0	N/A
control 15	1	Birth control (name not specified)	1	Birth control (name not specified)
control 16	0	N/A	0	
control 17	1	Birth control (name not specified)	1	Birth control (name not specified)
control 18	0	N/A	0	N/A
control 19	0	N/A	0	N/A
control 20	0	N/A	0	N/A
control 21	0	N/A	0	N/A
Patient 1	0	N/A	Not asked*	N/A
Patient 2	0	N/A	0	N/A
Patient 3	1	Methylphenidate, Amphetamine/Dextroamphetamine, Bupropion, Lamotrigine, Citalopram, Fluoxetine, Divalproex sodium	1	Citalopram, Lamotrigine, Iron Supplement
Patient 4	0	N/A	0	N/A
Patient 5	0	N/A	0	N/A
Patient 6	1	Oxcarbazepine	1	Oxcarbazepine, Levonorgestrel and Ethinyl Estradiol
Patient 7	1	Bupropion, Disulfiram	1	Bupropion
Patient 8	0	N/A	0	N/A
Patient 9	0	N/A	0	N/A
Patient 10	0	N/A	0	N/A
Patient 11	0		0	N/A

Patient 12	1	N/A	0	N/A
Patient 13	1	Birth control (name not specified)	1	Birth control (name not specified)
Patient 14	1	Aripiprazole, Sertraline, Lamotrigine, Ropinirole, Inhaler (name not specified)	1	Aripiprazole, Sertraline, Lamotrigine, Ropinirole
Patient 15	0	N/A	0	N/A
Patient 16	0	N/A	0	N/A
Patient 17	0	N/A	0	N/A
Patient 18	1	Sertraline, Aripiprazole	1	Aripiprazole
Patient 19	1	Oxcarbazepine	0	N/A
Patient 20	0	N/A	1	Acetaminophen, Dextromethorphan, Doxylamine
Patient 21	0	N/A	1	Ibuprofen
Patient 22	1	Antibiotics (name not specified)	1	Aripiprazole

*** The recency questionnaire for drug/medication use was later added to the study and therefore we do not have information of medication use on the day of MRI for 4 controls and 1 patient. However, it should be noted that the medication use for the most part did not differ between the off-site interview and on the day of MRI. Red indicates the subjects using some kind of medication.**
