# Supplementary material for: Female Adolescents with Severe Substance and Conduct Problems Have Substantially Less Brain Gray Matter Volume
Source: PLoS One. 2015 May 22;10(5):e0126368. doi: 10.1371/journal.pone.0126368 (PMC4441424; doi:10.1371/journal.pone.0126368)
Supplement: S2 Table — The region of interest (ROIs) where GM volumes in female controls exceeded volumes in SCP patients after excluding subjects on medication. (PDF) [file pone.0126368.s003.pdf]

Supporting Information S2 Table. Region of Interest analyses without the medicated subjects. Region of interest (ROIs) where GM volumes in female controls exceeded volumes in SCP patients after excluding subjects on medication. The table presents GM volume in each group for ROIs where controls>patients (in the whole-brain) after removing the subjects who were on medication at the initial interview. The table also includes total GM volume for each group.

	Controls (n=16)	Patients (n=13)	Reduction (%)	
**Significant Cluster (from the whole-brain analyses)**	**(means±sd)**	**(means±sd)**	**Patients**	**p-value***
**Cingulate Gyrus**	0.57±0.06	0.51±0.06	10.53	0.02
**Medial Prefrontal Cortex**	0.49±0.05	0.45±0.08	8.16	0.01
**Left Ventrolateral Prefrontal Cortex**	0.54±0.05	0.42±0.10	22.22	0.002
**Right Dorsolateral Prefrontal Cortex**	0.63±0.05	0.54±0.06	14.29	0.002
**Medial Orbitofrontal Cortex**	0.52±0.05	0.44±0.06	15.38	0.002
**Left Somatosensory Motor Cortex**	0.55±0.04	0.48±0.07	12.73	0.0007
**Left Angular Gyrus**	0.54±0.06	0.44±0.08	18.52	0.002
**Right Angular Gyrus**	0.61±0.03	0.52±0.06	14.75	0.0002
**Right Somatosensory Motor Cortex**	0.55±0.07	0.46±0.09	16.36	0.002
**Total GM Volume (ml)**	681.81±47.94	611.27±62.99	9.47	0.011

*Analyses of covariance: ROI as dependent variable and group, age and IQ as covariates.
